# Phalangeal fracture in a 60-year-old man after a low-voltages electric shock: A case report

**DOI:** 10.1097/MD.0000000000050279

**Published:** 2026-08-21

**Authors:** Yi Zhang, Jiaquan Yang, Kunqiang Chen, Song Zhou, Liangliang Zhou, Shiguo Gong, Ronghui Xie, Zhigang Zhou

**Affiliations:** a Department of Orthopedics, Jiujiang City Key Laboratory of Cell Therapy, Jiujiang No. 1 People’s Hospital, Jiujiang, Jiangxi, China.

**Keywords:** electric injury, electric shock, muscle contraction, orthopedic surgery, phalangeal fracture

## Abstract

**Rationale:**

Fractures caused by low-voltage electric shocks are extremely rare, especially those involving the distal appendicular skeleton. Most reported cases involve the proximal skeleton, such as the shoulder or vertebrae, and phalangeal fractures following low-voltage shocks without direct mechanical trauma have not been reported previously.

**Patient concerns:**

A 60-year-old man presented with persistent pain, swelling, limited thumb motion, and a non-healing ulcer after sustaining a 220V household electric shock to his left thumb. The patient reported falling onto his buttocks immediately after the shock but did not sustain any direct trauma to the injured thumb.

**Diagnoses:**

Initial physical examination revealed pain and restricted thumb mobility, accompanied by an electric burn. Twenty-five days later, an X-ray demonstrated a comminuted fracture of the left thumb phalanx with associated soft tissue injury.

**Interventions:**

The patient underwent stepwise surgical management, including debridement of necrotic tissue, bone cement filling, and subsequent interphalangeal joint fusion with Kirschner wire fixation. Perioperative antibiotics were administered according to microbial culture results.

**Outcomes:**

The wound healed well after staged surgeries. Radiographs at one month showed callus formation, and at 6 months follow-up, the patient regained pain-free thumb function adequate for daily activities with stable bone healing.

**Lessons:**

Although extremely rare, this case suggests that low-voltage electric shocks may occasionally cause fractures in the absence of direct trauma through tetanic muscle contraction. Clinicians should maintain a high index of suspicion for occult fractures in patients with localized finger pain after electric injuries and perform radiographic evaluation promptly to avoid missed diagnoses.

## 1. Introduction

Phalangeal fractures are among the most common fractures of the hand,^[1]^ typically resulting from direct or indirect trauma. However, rare etiologies such as electric shock-induced fractures have been documented,^[2–4]^ albeit predominantly in the context of high-voltage exposures or associated falls.^[5]^ While electric shock-related fractures are more frequently reported in proximal skeletal regions, distal appendicular involvement remains exceptionally uncommon. Importantly, fractures related to electrical injury are often associated with secondary trauma, such as falls or direct mechanical impact. Cases occurring without direct trauma to the affected limb are particularly rare. This report presents a rare case of a comminuted phalangeal fracture of the thumb following a low-voltage household electric shock, occurring without direct mechanical trauma to the thumb, despite the patient falling onto his buttocks after the incident. The aim of this report is to describe this unusual presentation, discuss its potential mechanism, and highlight the importance of early imaging in electric shock injuries to prevent delayed diagnosis.

## 2. Case report

**Figure 1.**
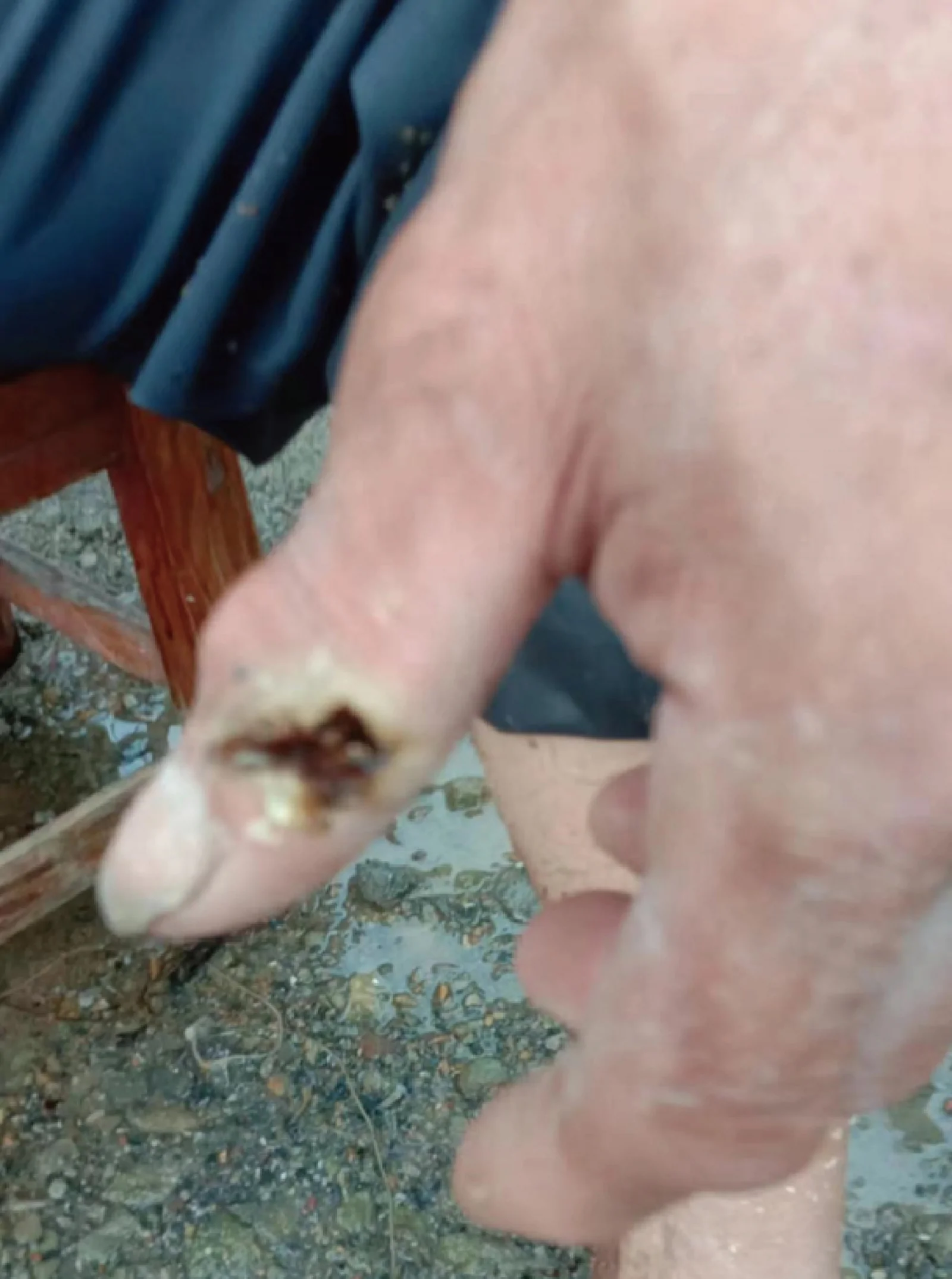
Electric mark on the left thumb.

A 60-year-old man presented to a local hospital immediately after suffering an electrical shock 26 days prior, with complaints of pain and electric marks on his left thumb (Fig. 1). His relatives reported that he fell on his buttocks after the shock and subsequently freed his left hand from the wire. He did not fall on his hands and did not experience any direct mechanical impact on the thumb. He did not lose consciousness. At the local hospital, examination revealed pain in the left thumb, limited movement, and electric marks, with no other abnormalities. The emergency physician attributed the limited thumb movement to pain, performed debridement, and discharged the patient.

**Figure 2.**
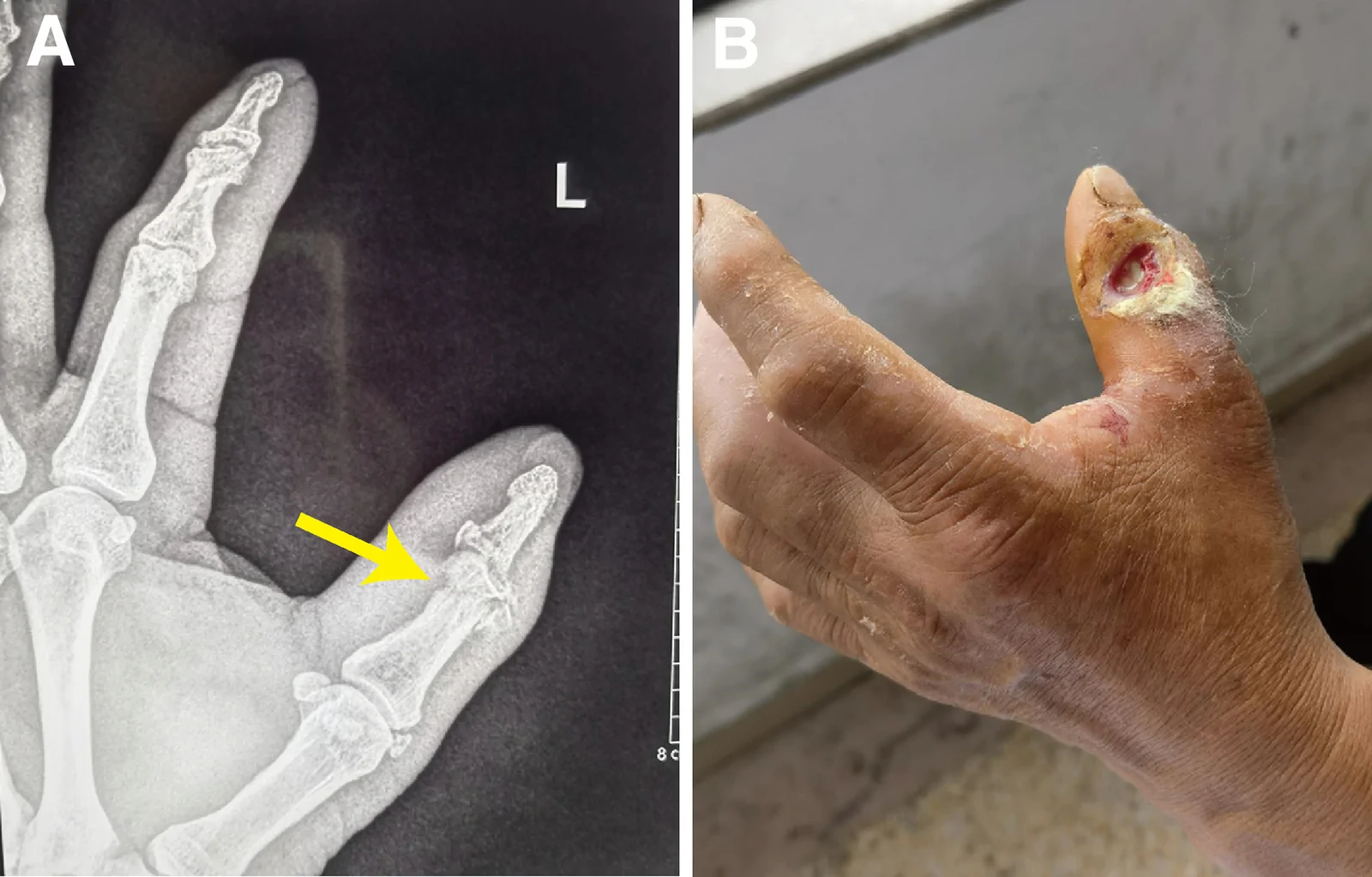
(A) X-ray showing a comminuted fracture of the left thumb phalanx (yellow arrow). (B) Skin ulcer on the medial side of the thumb.

After discharge, the patient continued to experience pain and limited mobility in his left thumb, with a persistent non-healing skin ulcer. He returned to the local hospital one day prior, where an X-ray revealed a comminuted fracture of the left thumb phalanx (Fig. 2A). Seeking further treatment, the patient presented to our hospital. Examination revealed tenderness at the interphalangeal joint, palpable bone crepitus, joint instability, limited movement, and a 1 cm diameter skin ulcer with visible necrotic tissue (Fig. 2B). Peripheral blood supply and sensation were normal.

**Figure 3.**
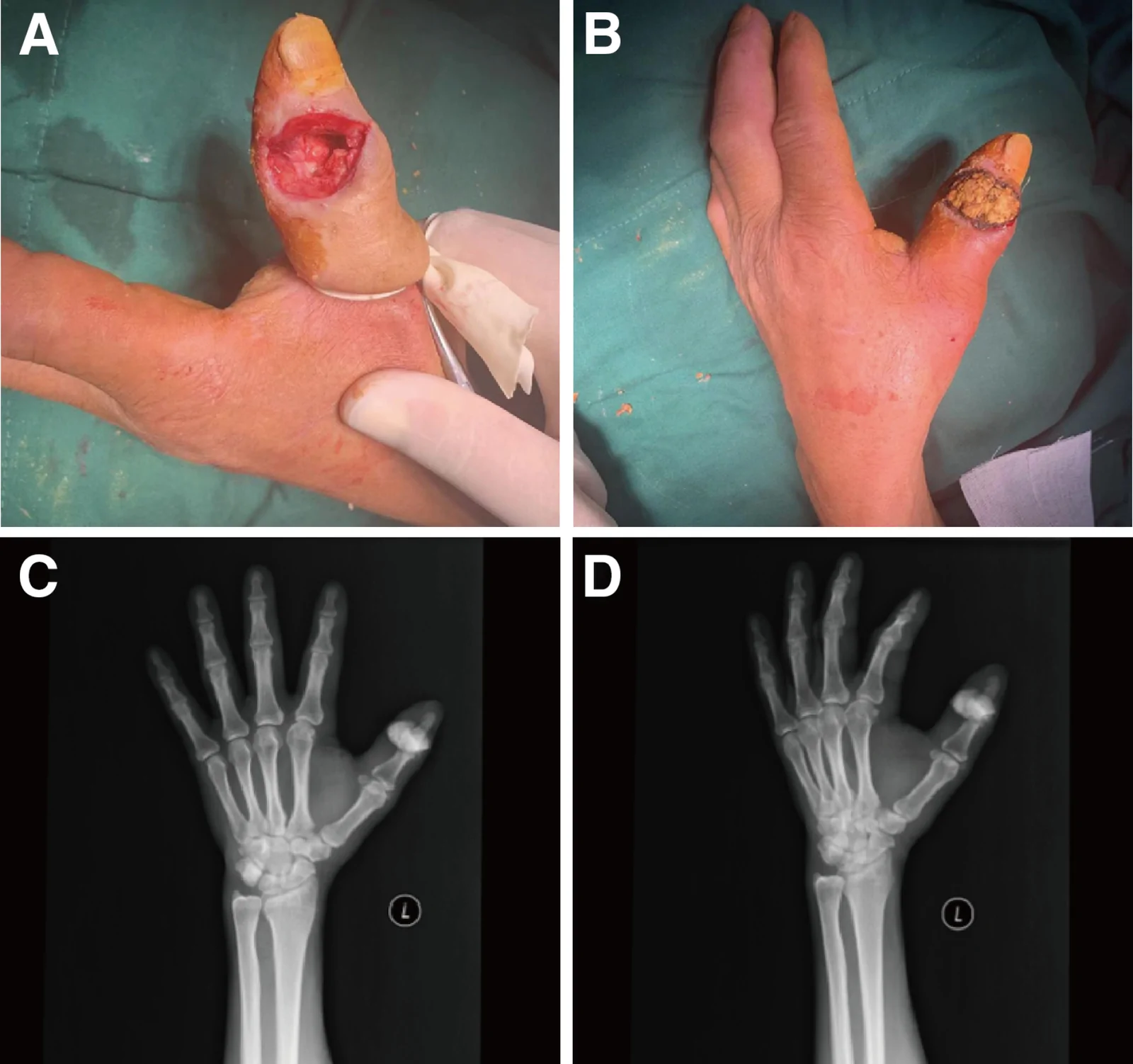
Preoperative (A) and postoperative (B) photos of debridement and bone cement filling treatment for necrotic tissue on the left thumb. Postoperative anteroposterior (C) and lateral (D) X-rays of the left hand.

**Figure 4.**
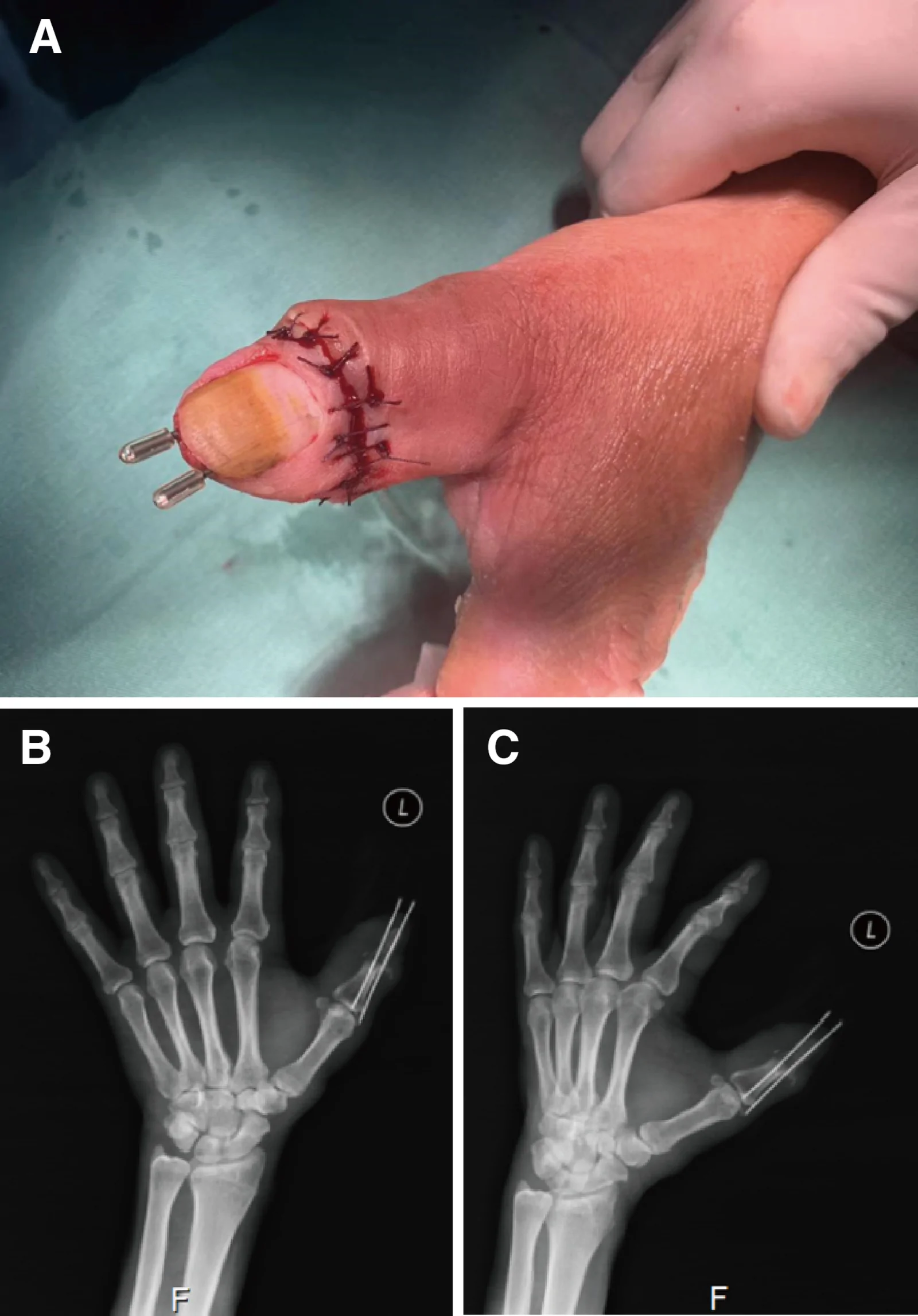
Postoperative photos (A) of bone cement removal and interphalangeal joint fusion with Kirschner wire internal fixation. Postoperative anteroposterior (B) and lateral (C) X-rays of the left hand.

**Figure 5.**
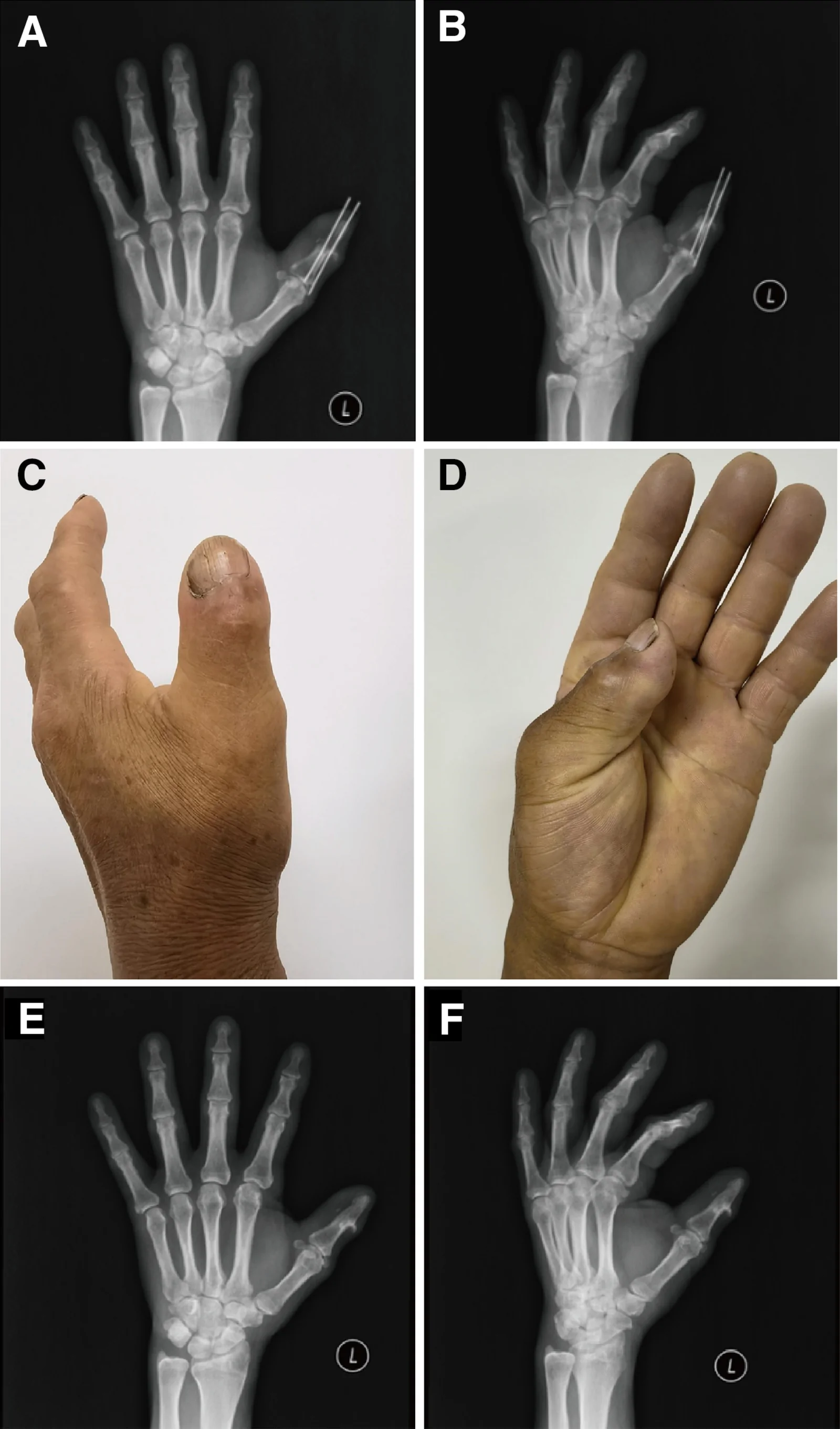
One-month postoperative anteroposterior (A) and lateral (B) X-rays of the left hand. Six-month postoperative photos (C,D) of the patient’s left hand. Six-month postoperative anteroposterior (E) and lateral (F) X-rays of the left hand.

A multidisciplinary evaluation by the orthopedics and infectious diseases departments was conducted. Wound secretions were sent for microbial culture; subsequently, the antibiotic therapy was adjusted according to the microbial culture results. Then, the patient underwent debridement of necrotic tissue and bone cement filling (Fig. 3A–D). This staged surgical approach was selected to remove necrotic tissue, control potential infection, and stabilize the soft tissue environment before definitive fixation. Postoperatively, the patient received levofloxacin. On the 18th postoperative day, bone cement removal and interphalangeal joint fusion with Kirschner wire fixation were performed (Fig. 4A–C). The wound healed well, and the patient was discharged on the 5th postoperative day. One-month follow-up X-rays showed callus formation (Fig. 5A and B). At 6 months, the patient’s left thumb function had returned to pain-free function sufficient for daily activities, with stable bone healing (Fig. 5C–F). A complete timeline is presented in Table 1.

**Table 1:** Timeline of clinical events.

Date/ Time (relative to injury)	Event & Findings	Interventions	Outcomes
Day 0 (injury)	220V low-voltage electric shock to left thumb; patient fell on buttocks but not on hands; electric burn on thumb; thumb pain and restricted motion	Local hospital: physical exam, wound debridement	Discharged the same day
Day 1–25	Persistent pain, limited thumb mobility, non-healing ulcer	Conservative wound care at home	No improvement
Day 25	Returned to local hospital; X-ray showed comminuted fracture of left thumb phalanx	Referred to our hospital	Diagnosis established
Day 26 (admission to our hospital)	Examination revealed skin ulcer with necrotic tissue, bone crepitus, joint instability	Multidisciplinary evaluation (orthopedics + infectious diseases)	Plan for staged surgery
Day 27	First operation: debridement, necrotic tissue removal, bone cement filling	Perioperative antibiotics guided by culture	Wound stabilized
Post-op Day 18	Wound dry, no exudate	Second operation: bone cement removal and interphalangeal joint fusion with Kirschner wire fixation	Stable wound healing
Post-op Day 23	Discharged from hospital	Outpatient follow-up planned	Stable
1 mo post-op	X-ray: callus formation	Removal of Kirschner wires	Continued recovery
6 mo post-op	Follow-up exam and X-rays	No intervention needed	Full functional recovery of left thumb

post-op = post-operative, V = voltage.

## 3. Discussion

Electrical injuries can cause a spectrum of musculoskeletal complications, ranging from burns to fractures.^[6]^ Fractures following electric shock are typically attributed to high-voltage currents or secondary trauma such as falls.^[7,8]^ However, rare cases of low-voltage-induced fractures without associated trauma have been reported, primarily involving the shoulder, vertebrae, or forearm.

There are few reports in the literature of fractures occurring after low-voltage electric shocks without other indirect trauma.^[9–12]^ Stone et al conducted a case-based review of 21 publications, summarizing the anatomical distribution of fracture sites in 22 cases of electric shock-induced fractures.^[13,14]^ The most common fractures were shoulder fractures (8 cases of proximal humerus fractures and 7 cases of scapula fractures), followed by forearm fractures (1 case of Colles’ fracture, 1 case of Galeazzi fracture, 1 case of greenstick fracture, and 1 case of distal radius fracture). The mechanism of these fractures is often due to the tetanic contraction and pulling of the bones at the attachment points of muscles stimulated by the electric current, leading to fractures. Additionally, another study^[9]^ reported a 22-year-old man who suffered multiple thoracic vertebral fractures following a low-voltage electric shock. After experiencing a brief whole-body spasm due to an electric guitar shock, the patient disconnected the power source and escaped the electric shock. Throughout the incident, the patient remained conscious and did not fall. He complained of pain in the scapular area, and subsequent X-rays revealed compression fractures of the T3 to T5 thoracic vertebrae.

Another noteworthy aspect of this case is the delayed diagnosis of the fracture. The injury was initially attributed to electrical burn-related pain and soft tissue injury, which may have masked the underlying skeletal damage. In addition, fractures caused by low-voltage electrical shocks are extremely rare, and the absence of obvious deformity at the initial presentation may have reduced clinical suspicion. These factors likely contributed to the delayed radiographic evaluation and diagnosis. This case therefore highlights the importance of considering occult fractures when patients present with persistent localized pain following electrical injury.

Our reported case involves a comminuted fracture of the thumb phalanx following a 220V shock – a distal site rarely reported in this context. This type of distal appendicular skeletal fracture is extremely rare. The most plausible mechanism is localized tetanic muscle contraction induced by electrical stimulation. Concentration of the electrical current at the thumb may have triggered sudden contraction of the intrinsic and extrinsic thumb muscles, generating sufficient force to fracture the phalanx at a structurally vulnerable site. Although the muscles surrounding the thumb are relatively small compared with those of the shoulder girdle or spine, the rapid and involuntary nature of tetanic contraction can produce substantial transient forces. Other contributing factors, such as potential microstructural bone vulnerability, may also have played a role.

## 4. Conclusion

Phalangeal fractures due to low-voltage electric shock are exceedingly rare and have not been previously reported. This case suggests that, although uncommon, low-voltage electrical exposure may occasionally induce fractures through tetanic muscle contraction even in distal skeletal regions. Clinicians should therefore consider the possibility of occult fractures in patients presenting with persistent digital pain after electrical injury and pursue timely radiographic evaluation to prevent missed diagnoses.

## Author contributions

**Conceptualization:** Liangliang Zhou, Ronghui Xie, Zhigang Zhou.

**Data curation:** Jiaquan Yang.

**Investigation:** Yi Zhang, Kunqiang Chen, Song Zhou.

**Supervision:** Ronghui Xie.

**Writing – original draft:** Yi Zhang.

**Writing – review & editing:** Shiguo Gong, Ronghui Xie, Zhigang Zhou.
